# Spatially fractionated minibeam radiation delivered at clinically feasible dose rates induces transient vascular permeability

**DOI:** 10.1038/s41598-025-87395-9

**Published:** 2025-03-10

**Authors:** Jennifer Fazzari, Cristian Fernandez-Palomo, Paolo Pellicioli, Liam Day, Verdiana Trappetti, Fabrice Lucien-Matteoni, Yohan Kim, Robert Mutter, Sean Park, Michael Grams, Valentin Djonov

**Affiliations:** 1 Institute of Anatomy, University of Bern, Bern, Switzerland; 2 ID17 Biomedical Beamline, European Synchrotron Radiation Facility, Grenoble, France; 3 Department of Urology, Mayo Clinic, Rochester, MN USA; 4 Department of Radiation Oncology, Mayo Clinic, Rochester, MN USA; 5 Department of Pharmacology, Mayo Clinic, Rochester, MN USA

**Keywords:** Oncology, Preclinical research

## Abstract

Microbeam Radiation Therapy is a preclinical form of spatially fractionated radiation therapy that utilizes synchrotron X-rays to deliver highly heterogeneous dose distributions at a micrometric scale. This radiation scheme has been shown to facilitate the induction of controlled and reversible vascular permeability, enhancing treatment efficacy of systemic therapeutic agents. Despite the promising preclinical results, translating microbeam SFRT to the clinic has been hindered by a reliance on synchrotron sources that operate at dose rates orders of magnitude greater than what is possible with clinical machines. Without rapid dose delivery, the microbeam geometry is susceptible to blurring due to physiologic motion when delivered at clinical dose rates. Therefore, larger beam widths, spaced further apart (minibeams) were employed to determine whether such effects can be observed with clinically achievable doses and dose rates. Vascular permeability was assessed in the chick chorioallantoic membrane vasculature following minibeam irradiation delivered at peak doses (10 Gy) and dose rates (10 Gy/s and 0.05 Gy/s) approaching clinical relevance. Transient, reversible permeability could be induced at these dose rates beginning 1–2 h post-irradiation. This was followed by temporary vascular occlusion in the beam path that was resolved by 7 h when delivered at 10 Gy/s but persisted longer when delivered at 0.05 Gy/s. Despite these changes, vascular function was maintained at both dose rates by 24 h post-IR, differing only in the degree of regeneration. The induction of permeability was also maintained when using a clinical orthovoltage system further supporting the potential clinical application of minibeam radiation therapy.

**Supplementary Information:**

The online version contains supplementary material available at 10.1038/s41598-025-87395-9.

## Introduction

Inducing vascular permeability is a therapeutic strategy designed to improve drug penetration within tumors. The complex characteristics of tumor vasculature and high interstitial pressure hinder the accumulation of therapeutic concentrations from systemically administered agents. Overcoming this obstacle has the potential to significantly enhance the efficacy of systemic therapy such as chemo- and immunotherapy^1^. Early radiation studies describe the rapid increase in blood vessel permeability following exposure to single doses of homogenous radiation^2–4^ with different radiation modalities and schemes having differential effects on the integrity of the normal and tumor microvasculature^5,6^. Acute episodes of vascular permeability following large, single doses are associated with endothelial cell destruction and/or opening of intercellular junctions^7^ with its onset and duration being inversely dose-dependent^8^. These changes can be progressive resulting in microvessel rarefaction and passive hyperpermeability^9–11^, which are detrimental to normal tissue integrity^12^.

Microbeam Radiation Therapy is a preclinical form of spatially fractionated radiation therapy (SFRT) known for its exceptional normal tissue sparing capacity (reviewed by Fernandez et al.^13^) allowing for the delivery of doses orders of magnitude higher than conventional radiation therapy. This has been attributed to microbeam SFRT’s heterogenous, micrometric dose distribution, consisting of high “peak” dose regions (> 100 Gy) interspaced by low “valley” dose regions. The width of the peak-irradiated regions typically ranges from 25 to 100 μm with a center-to-center spacing of 50 to 500 μm. This pattern of peak and valley dose delivery has been shown to preferentially destroy immature, tumor-like vessels^14,15^ while preserving mature vessels of healthy tissues^16,17^. In addition, the microbeam geometry has been shown to induce transient vascular permeability in peak regions without compromising vascular function^14,18,19^. This has been exploited to enhance drug delivery to solid tumors and has been shown to improve treatment response when combined with systemic therapy^20,21^. Microbeam SFRT is typically administered using ultra-high dose rate synchrotron sources (kGy/s), which permits the delivery of these highly collimated microbeams while maintaining a high peak-valley dose differential and avoid blurring from physiologic motion. The requirement for ultra-high dose rates has hindered its clinical implementation as clinical irradiators operate at much lower dose rates, which could result in blurring of MRT geometry and consequently, reduce its normal tissue sparing capacity. This in turn, prevents delivery of the typical microbeam geometry outside of a synchrotron. Although the therapeutic benefit of microbeam SFRT, including its ability to induce transient vascular permeability, has been extensively described in a preclinical setting, it is unknown whether these microbeam-specific parameters (kGy/s dose rates, micrometric geometry, peak doses > 100 Gy) are essential for inducing theses effects or whether they could be adapted to radiation schemes that can be accommodated by clinical irradiators. Minibeam SFRT was developed to address some of these concerns by employing wider beams (≥ 500 μm) spaced further apart (≥ 1000 μm) in order to maintain clearly delineated peak and valley dose regions when delivered at lower dose rates. Although it has shown to maintain some of the biological effects of microbeam SFRT, its effect on vasculature has yet to be described. We therefore investigated how microSFRT-induced vascular permeability is impacted by a wider beam geometry (0.5 mm beams spaced 1 mm center-to-center), lower peak doses (10 Gy) and a significantly reduced dose rate (10 Gy/s and 0.5 Gy/s). These encompass radiation parameters that are feasible with non-synchrotron sources including those available in the clinic. This study reports for the first time that minibeam SFRT induces a transient vascular permeability window similar to that of microbeam SFRT without ultra-high dose rates. This demonstrates that minibeam SFRT is a viable option for translating the unique biological effects of microbeam SFRT to the clinic where it could be employed to improve the accumulation of systemic agents in solid tumors.

## Materials and methods

### Chick chorioallantoic membrane model

We employed the chicken chorioallantoic membrane (CAM) model to screen vascular changes and induction of permeability in real-time. The CAM is the extraembryonic membrane of the developing chick embryo and has commonly been used to study vessel dynamics and hemodynamic properties in a qualitative and quantitative manner^22^. The CAM has been widely applied as a model of normal vascular growth and remodeling processes in the field of angiogenesis^23^ and wound healing^22^, and has been instrumental in describing the vascular effects induced by microbeam SFRT^14,20^. This vascular model was therefore selected to investigate whether minibeam radiation delivered at peak doses and dose rates orders of magnitude below those used in microbeam SFRT could induce transient vascular permeability with a potential for future clinical translation.

Fertilized leghorn chicken eggs obtained from a commercial hatchery were cleaned with 70% ethanol and incubated at 37 °C for 72 h prior to opening. As per the shell-free culture method previously described^24^, on embryonic development day (ED) 3, eggs were transferred to a sterile petri dish and returned to 37 °C.

### Synchrotron minibeam irradiation

On ED 10, the CAM of the developing embryos were irradiated with minibeam SFRT at the European Synchrotron Radiation Facility’s (ESRF) ID17 biomedical beamline, Grenoble, France. The irradiation field consisted of an array of 11 minibeams, 0.5 mm wide and 2 mm high, each spaced by 1 mm center-to-center. The spatial fractionation of the homogenous X-ray beam generated by the synchrotron source was achieved using a multislit collimator, which is a machined block of metal with equidistant apertures. The use of the multislit collimator allows for the delivery of all beamlets simultaneously. Expanding the typical microbeam geometry (0.05 mm beams, 0.4 mm center-to-center spacing) to that of minibeams (0.5 mm beams, 1 mm center-to-center spacing) was tested as it is a more feasible irradiation scheme for currently existing clinical orthovoltage machines.

The SFRT field was delivered through the side of the petri-dish into the CAM as shown in Supplementary Fig. S2. The alignment of the CAM surface within the 2 mm high synchrotron beam was achieved using cameras positioned relative to the sample and X-ray beam. The definition of fiducial markers on the monitor, established before each experimental session and during beam preparation, allows for the alignment of the CAM’s liquid surface within the beam with a precision of approximately ± 100 μm. To avoid missing the upper layer of the CAM, the beam was directed 500 μm above the liquid-air surface, ensuring that a 1.5 mm high beam intersected the CAM.

Two dose rates of 10 Gy/s and 0.05 Gy/s were employed to represent both achievable dose rates of newly developed preclinical^25–27^ and clinical irradiators, respectively. The synchrotron was operating at an average current of 69 mA with photon mean energies of 161.5 keV and 197.5 keV after filtration for the 10 Gy/s and 0.05 Gy/s dose rates, respectively. Peak depth-dose profiles through the CAM were calculated using Geant4 (ver. 10.6.01) Monte Carlo simulations. The ex-ovo CAM was modelled in Geant4 as a disk of water enclosed by a layer of polystyrene for the petri-dish. From the simulated peak depth-dose profiles, an exposure time was calculated to deliver a peak dose of 10 Gy at 20 mm depth in the CAM for both dose rates. The corresponding Monte Carlo calculated valley doses were 0.216 Gy and 0.296 Gy for the 10 Gy/s and 0.05 Gy/s dose rates, respectively (Supplementary Fig. S1A). This difference in the delivered valley dose is attributed to the varying photon spectrum energies associated with the two dose rates. The spectrum with the higher mean energy contains photons that scatter more when passing through the target, and secondary radiation travels further within the target material before thermalizing. Both factors contribute to a higher dose delivery to the valley region for the spectrum associated with the lower dose rate. An overview of the experimental set-up and calculated peak depth-dose profiles is shown in Supplementary Fig. S2. Radiation parameters are summarized in supplementary Table 1.

### Clinical minibeam irradiation

Clinical minibeam irradiation was delivered using an Xstrahl 300 orthovoltage radiation therapy machine (Xstrahl Inc. Georgia, USA) operating at 180 kVp, with a corresponding central-axis mean photon energy of approximately 75 keV after filtration. The beam was collimated with a 30 mm diameter cone and subsequently spatially fractionated with a 2.5 mm thick tungsten collimator delivering 0.5 mm beams spaced 1.1 mm center-to-center. The collimator was held in place by a custom-made 3D printed holder attached directly to the cone 30 cm from the source. The beam was delivered from above through the top of the CAM. A peak dose of 10 Gy at a dose rate of 0.02 Gy/s was delivered to the surface of the CAM with a corresponding valley dose of 1.35 Gy. The peak and valley doses were determined with EBT3 radiochromic films (Ashland Advanced Materials, Bridgewater, NJ, US) using a methodology previously described^28^. Briefly, films were scanned using an Epson 12000XL scanner at 1200 dpi resulting in a resolution of 0.02 mm. Peak and valley doses were obtained as mean doses along a 0.2 mm × 10 mm wide line profile centered on each of the 10 central peaks and adjacent valley regions. Three independent film measurements were made resulting in 30 central peak and valley dose measurements, which were then averaged to give a single representative peak and valley dose. A representative dose profile is depicted in supplementary Fig. S1B. Radiation parameters are summarized in supplementary Table 1.

### Assessment of vascular permeability: intravital microscopy

Vascular permeability was assessed at various timepoints: 1 h, 2 h, 7 h and 24 h post-IR for synchrotron irradiations and at 2 h and 24 h post-IR with the orthovoltage source. Immediately prior to observation, 100 µl of 2.5% FITC dextran (150 kDa) in saline was injected via an artery distant from the irradiation path. For synchrotron irradiations, CAM vasculature was then observed with a Zeiss Axioscope fluorescent microscope (Zeiss AG, Oberkocken, Germany) using a 450–490/515–565 nm emission/excitation filter (Zeiss Filter Set 10) and recorded with a monochromatic industrial camera (Baumer model VCXU-50 M; Baumer International GmbH, Stockach, Germany) controlled by Baumer Camera Explorer v3.3.0 software. For orthovoltage irradiations, images were acquired using a Nikon SMZ stereo microscope equipped with a Digital Sight Ri2 camera (Nikon Instruments, New York, USA). Due to toxicity of prolonged exposure to FITC dextran, timepoints could not be assessed in the same CAM and individual CAMs were irradiated for each timepoint. Five CAMs were irradiated per dose rate and timepoint.

For the CAMs irradiated at the synchrotron facility, the irradiation path (including the entrance and exit of the beam) was marked on the petri dish and observations were made at the same distance from the entrance, where peak doses were 10 Gy as determined by Monte Carlo simulations (Supplementary Fig. 2B).

### Tissue collection

At each given timepoint post synchrotron-IR, the irradiated CAM was fixed with Karnovsky solution (2.5% glutaraldehyde, 2% PFA in 0.1 M sodium cacodylate buffer, pH 7.4) applied on top of and injected under the CAM in the irradiated region. After approximately 2 h, the region of interest (2 cm from the marked entrance) was removed from the CAM with directionality indicated to ensure orientation of the beam path was maintained. The tissue was photographed and then placed into an Eppendorf tube containing Karnovsky solution. Samples were refrigerated until processing for transmission electron microscopy (TEM).

### Image processing

Due to the movement of the CAM during live imaging, image deconvolution was performed when necessary using Huygens Deconvolution Software (Version 1.54e, Scientific Volume Imaging B.V., Hilversum Netherlands) to enhance the quality of the acquired images.

To enhance the tubular structures present in our images we utilized the 3D line enhancement filter ImageJ plugin named “Tubeness”, which enhances line structures of various widths and removes non-line features and noise/artifacts such as those attributed to permeability of the FITC-dextran. This plugin employs a method based on the Hessian matrix to highlight linear structures, such as tubules, blood vessels, or other elongated features in the image^29^. The “Tubeness” filter was applied to the preprocessed images. The sigma parameter was adjusted to optimize the detection of tubular structures based on vessel diameter.

### Transmission electron microscopy (TEM)

Tissue blocks were washed 3 times in 0.1 M sodium cacodylate buffer (pH 7.4, 340 mOsmol/kg H_2_O) and post-fixed in 1% OsO_4_ (in 0.1 M sodium cacodylate buffer pH 7.4, 340 mOsmol/kg H_2_O). After another series of washings in 0.1 M sodium cacodylate buffer (pH 7.4, 340 mOsmol/kg H_2_O) tissue was dehydrated by an increasing concentration series of ethanol as follows: 70%, 80%, 90%, 96% for 30 min at each concentration and finally twice in 99% and 100% for 15 min each. Lastly, ethanol was replaced by 100% acetone. Finally, specimens were embedded in epoxy resin. Semithin sections of 1 μm were stained with Toluidine Blue and evaluated using a Zeiss M2 Light Microscope. Ultrathin sections of 70 nm thickness were cut using a Reichert Ultracut S microtome (Leica, Wetzlar Germany) and images were obtained using a Tecnai Spirit Bio Twin electron microscope (FEI, Oregon, USA).

### Animal ethics

Chicken embryos were not used past ED 14 and therefore did not require institutional ethics approval in accordance with the European Directive 2010/63/EU, Swiss Cantonal Veterinary Authorities and the US National Institutes of Health’s Office of Laboratory Animal Welfare. The study was carried out in compliance with the ARRIVE guidelines^30^.

## Results

### Synchrotron minibeam irradiations at 10 Gy/s

**Fig. 1:**
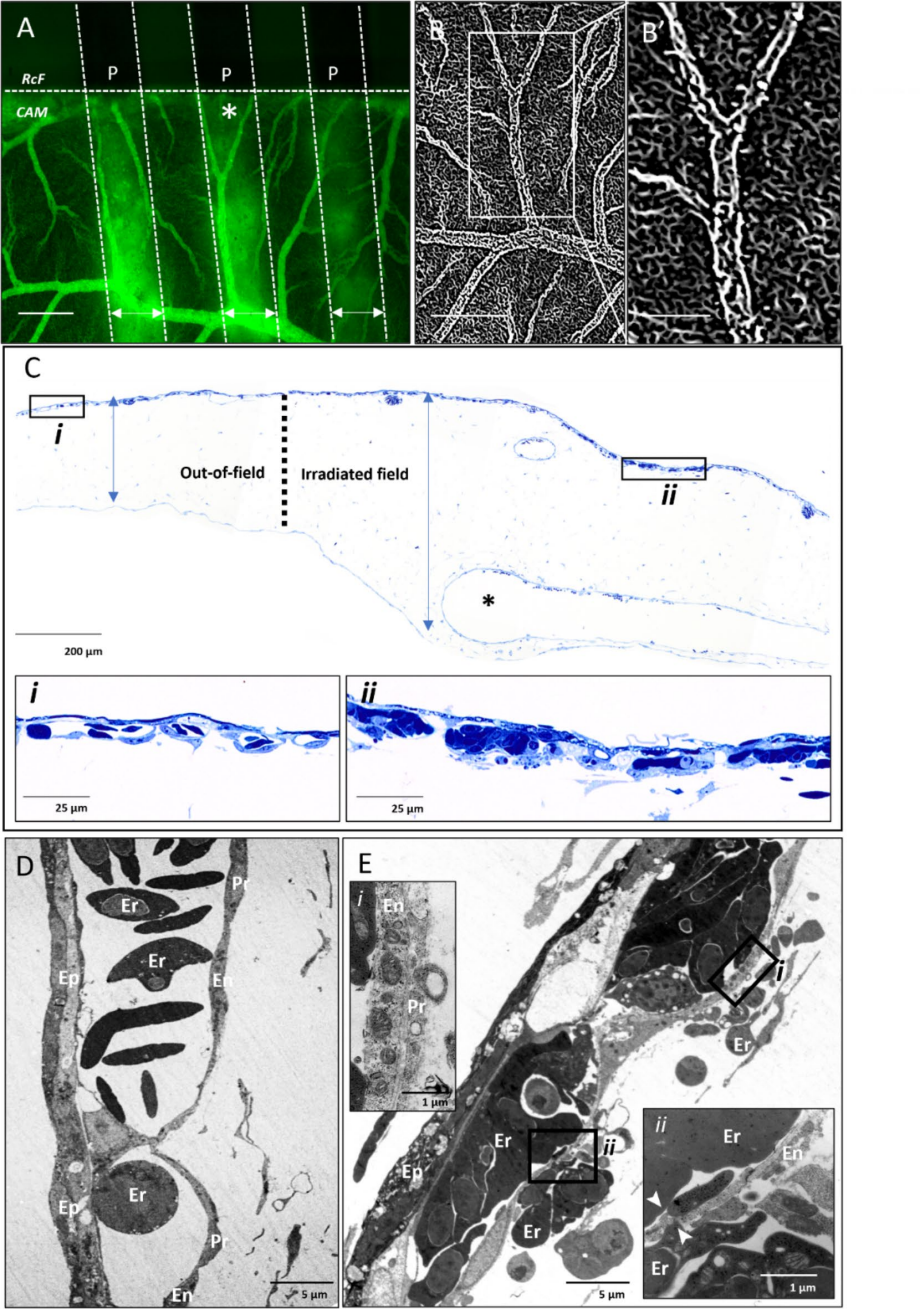
Vascular permeability induced 1 hour following delivery of 10 Gy peak dose at 10 Gy/s. **(A)** Permeability of FITC-dextran (150 kDa) is observed as diffuse fluorescence in peak-irradiated regions (P, double-headed arrows) as confirmed by the presence of radiochromic film (*RcF*). **(B)** Vascular connectivity from one peak region in A (asterisk) was examined using vessel feature detection revealing intact microvasculature in both peak and valley regions at this time point and shown at a higher magnification in (B’). **(C)** Semithin section of CAM displays dilation of mesenchyme (edema indicated by blue double arrowheads) in irradiated region relative to out-of-field region with capillary plexus in out-of-field region appearing intact (C*i*) while those in the irradiation field are densely congested with erythrocytes and occluded by swelling epithelium (*Cii*). **(D)** Ultrastructure of vasculature in the out-of-field region is comprised of elongated, continuous, and tightly associated endothelial (En) and peri-endothelial (Pr) cells as well as a homogenous epithelium (Ep). **(E)** Ultrastructure of in-field vasculature reveals congested capillaries with signs of endothelial (En) and peri-endothelial (Pr) cell disintegration (*i*) as well as erythrocyte extravasation across the endothelial gaps (*ii;* arrowheads). Scale bar in A and B is equivalent to 500 μm, 250 μm in B’ and 1 μm in Ei and Eii.

Minibeam SFRT irradiation of the CAM resulted in a sequence of hemodynamic changes that were dose-rate dependent. One hour following synchrotron minibeam irradiation at 10 Gy/s, vascular permeability was observed as exudation of FITC-dextran (150 kDa) localized in the peak regions as confirmed by the increase in optical density (darkness) of the radiochromic film (Fig. 1A). Enhancement of vascular tubular structures in the images indicated that plexus integrity was maintained in both the peak and valley regions at this time point (Fig. 1B). An increase in overall CAM thickness in the irradiated field was observed as expansion of the mesenchyme corresponding to dilation of larger veins and edema stemming from increased vascular permeability (Fig. 1C). Microscopic changes in the capillary plexus were also visible at this timepoint in comparison with normal capillaries in the out-of-field region (Fig. 1C*i*) with those in the irradiated region showing endothelial cell blebbing and hemoconcentration (Fig. 1C*ii*). These changes in the irradiated region were observed in more detail at the ultrastructural level where a conserved vascular arrangement shows close inter-endothelial and peri-endothelial contacts (Fig. 1D) while irradiated regions had an electron dense and distended epithelium with neighboring capillaries containing gaps and fenestrations in the endothelium as well as disintegration of peri-endothelial cells (Fig. 1E*i*) and leakage of erythrocytes into the mesenchyme (Fig. 1E*ii*).

**Fig. 2:**
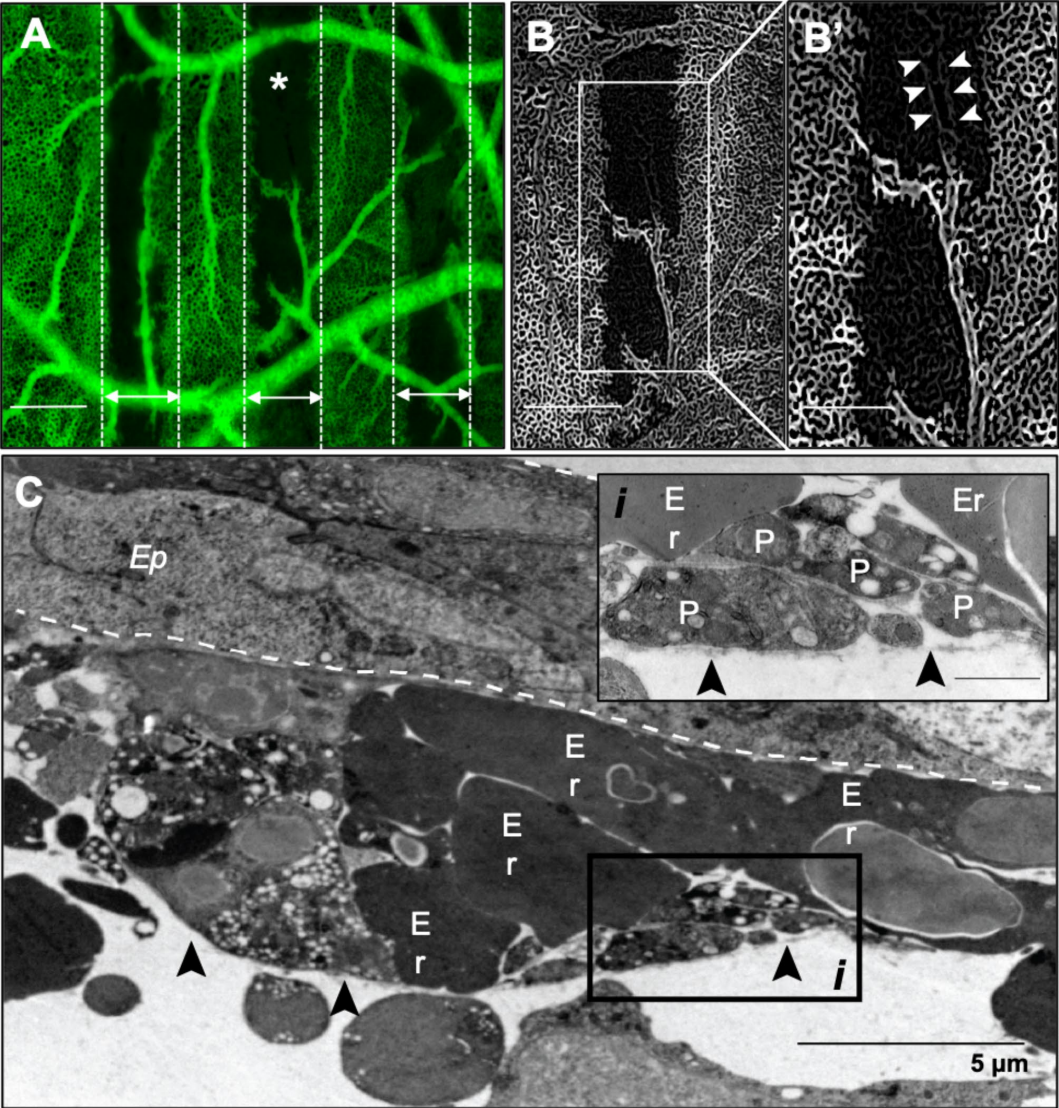
Vascular permeability in beam path ceases 2 hours after delivery of 10 Gy peak dose at 10 Gy/s. **(A)** Perfusion in the beam path ceases by 2 h post-irradiation and the capillary plexus in this region is unperfused clearly demarcating the peak (double-headed arrows) and valley regions. **(B)** Line enhancement filter was applied to one peak region selected from A (A; asterisks) to enhance visualization of vascular structure. Larger supplying vessels bridging the peak area and their attached microvessels remain perfused in the beam path. Complete irradiation of a larger vessel running longitudinally in the beam path appears partially occluded and is observed as an unperfused vascular structure (B’; arrowheads). **(C)** Ultrastructural changes show grossly enlarged epithelium and capillary structures with a basement membrane denuded of endothelial and peri-endothelial cells (black arrowheads). The vessel is completely congested with erythrocytes (Er) and platelets (P) with the latter accumulating along the exposed basement membrane (*i*). Scale bar in A and B is equivalent to 500 μm, 250 μm in B’ and 1 μm in Ci.

By 2 hrs post-IR permeability to the FITC-dextran tracer ceased with a loss of perfusion in the peak region apart from larger supplying vessels (Fig. 2A). Perfusion was, however, maintained in the valley regions. Enhancement of vascular features revealed that vascular structures were still present in the unperfused capillary plexus (Fig. 2B). Occlusion of a larger vessel that ran longitudinally in the beam path could be observed after application of the line enhancement filter where the vascular structure was visible but was not perfused with FITC-dextran (Fig. 2B*’; arrowheads*). Vessel occlusion was confirmed at the ultrastructural level with hemoconcentration observed in some capillaries in the irradiated region (Fig. 2C). Vessels had detached endothelial and peri-endothelial cells from the basement membrane on the mesenchymal side and in some instances the basement membrane was completely exposed (Fig. 2C; arrowheads) with an accumulation of platelets along these denuded regions (Fig. 2C*i*). Ultimately, the congestion of erythrocytes and platelets in the vessel along with a grossly expanded epithelium contributed to blood flow stasis and the absence of perfusion in the beam path.

**Fig. 3:**
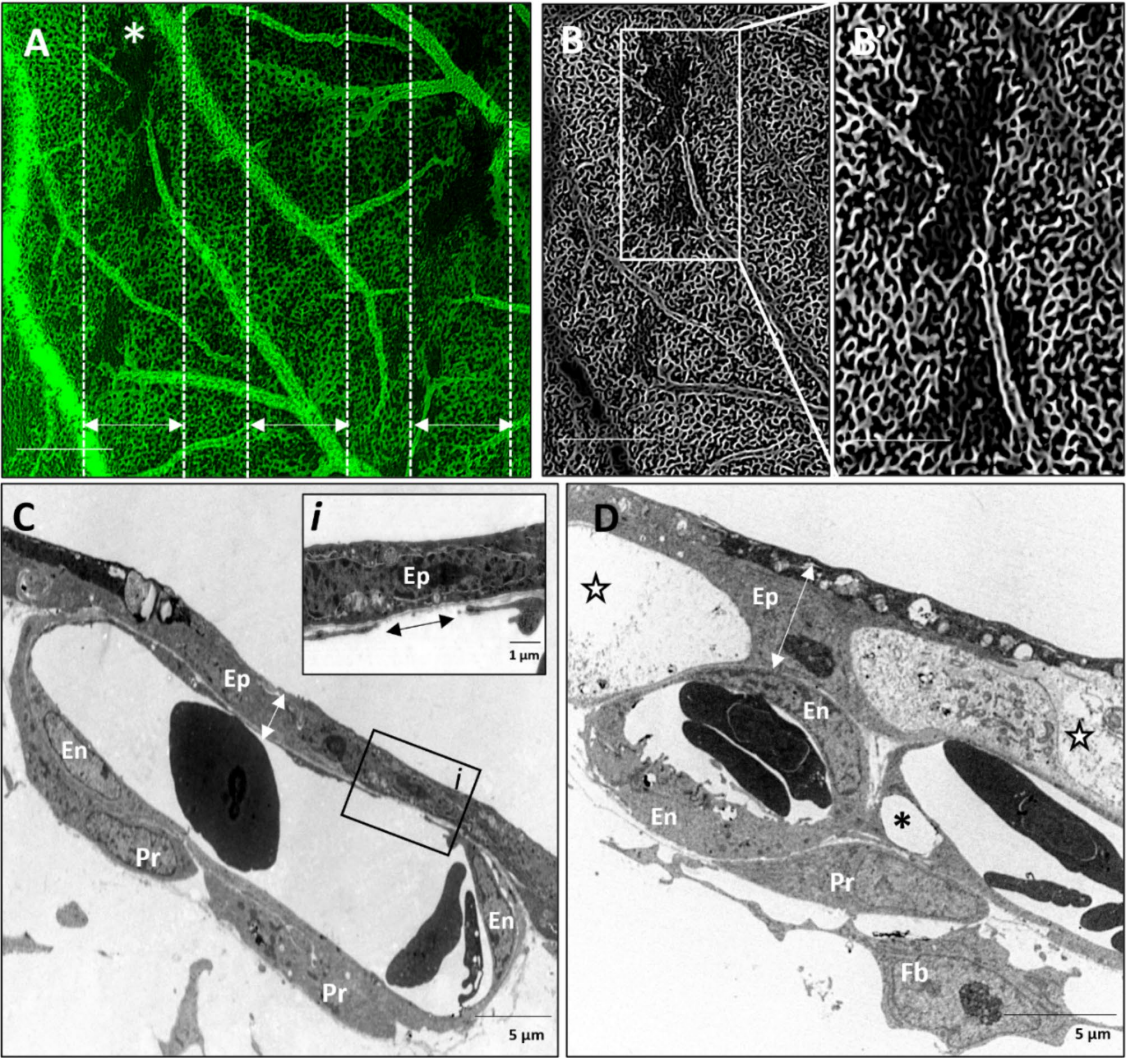
Perfusion of microvasculature is restored in majority of beam path 7 hours after minibeam irradiation with 10 Gy peak dose delivered at 10 Gy/s. **(A)** Perfusion is restored in most of the peak-irradiated microvasculature (double-headed arrows) with only sparse regions remaining unperfused. **(B)** Enhancement of vascular structures in one beam path selected in A (A; asterisk) shows a partial absence of vessel connectivity in these regions with vascular structures still detectible in the unperfused areas **(B’)**. **(C)** Ultrastructural changes reveal some CAM regions with electron dense epithelium (Ep) and capillaries showing signs of endothelial cell (En) recovery by migration of endothelial cell philopodia (double arrowhead along denuded basement membrane) (*i*) as well as dense peri-endothelial cell (Pr) coverage. **(D)** Other regions show enlarged, electron-lucent epithelium (stars). Capillaries in these regions show signs of regeneration with activated endothelial cells (En) exhibiting microvilli-like extensions into the lumen, many organelles and a rounded pericyte (Pr) partially attached to fibroblast-like cells (Fb) in close proximity to capillaries. Scale bar in A and B is equivalent to 500 μm and 250 μm in B’.

Vascular occlusion resolved in the majority of the peak-irradiated regions by 7 hrs post-IR, with only small, occluded zones remaining (Fig. 3A). In unperfused regions, connection to a large supplying vessel was absent (Fig. 3B, B’). Patterns of differential epithelial and endothelial changes were observed at this timepoint corresponding to changes in epithelial thickness as well as endothelial recovery. Vessel recovery was observed as both an expansion/migration of existing endothelial cells adjacent to electron dense epithelium of reduced thickness (Fig. 3C). This endothelial expansion was characterized by filopodia-like extensions of endothelial cells bridging a gap over the denuded basement membrane (Fig. 3C*i*). In other regions, early stages of endothelial regeneration were characterized by enlarged organelle-rich endothelial cells with microvilli-like extensions into the vessel lumen as well as enlarged and rounded peri-endothelial cells in close proximity without tight attachment to endothelial cells (Fig. 3D). These vascular changes coincided with regions exhibiting epithelial swelling characterized by large electron lucent regions.

**Fig. 4:**
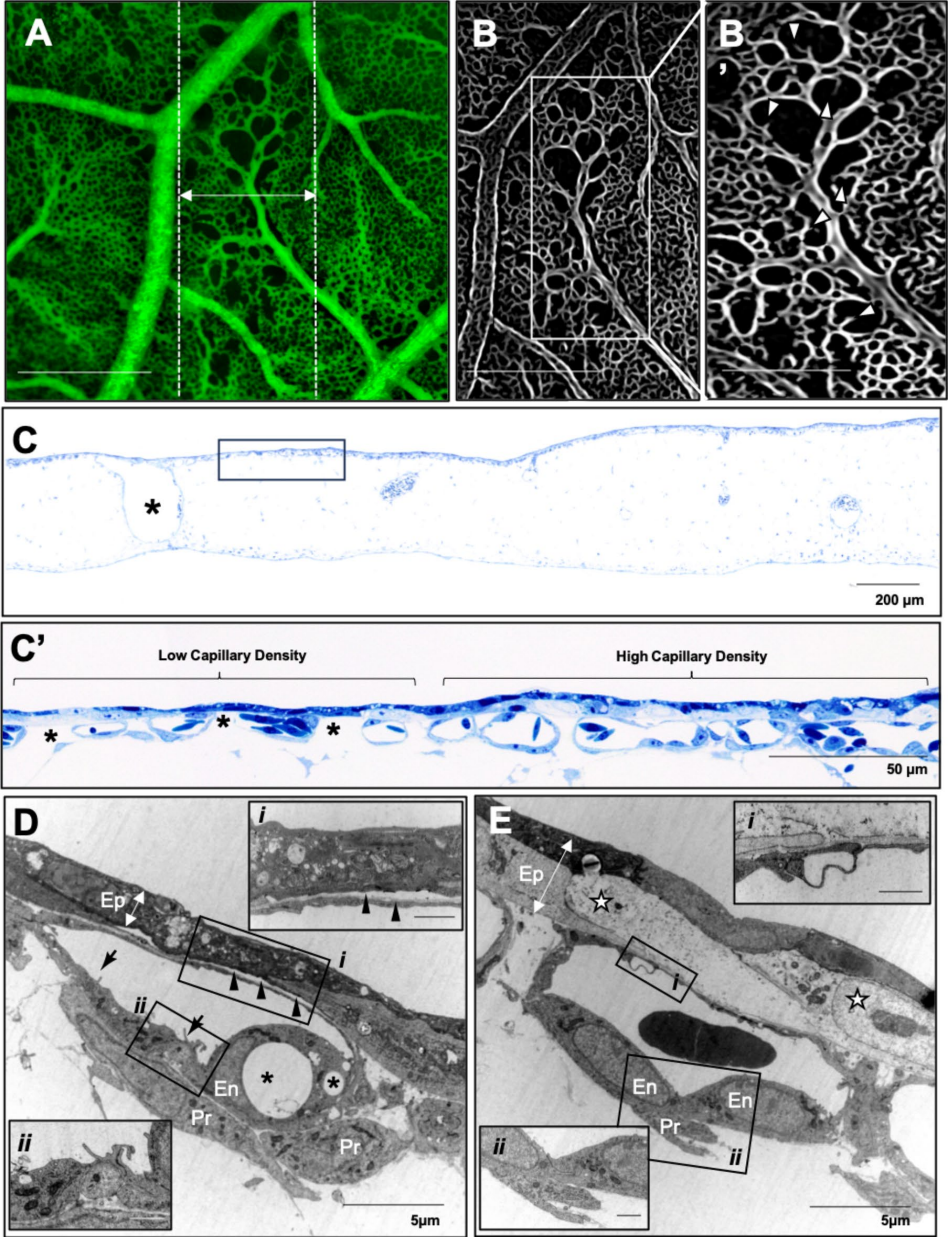
Signs of microvasculature regeneration are observed in the beam path 24 hours after delivery of 10 Gy peak dose minibeams delivered at 10 Gy/s. **(A)** By 24 hours post-irradiation there is almost complete restoration of perfusion across the beam path (dotted lines) with only a sub-500 μm region having large gaps **(B)** Vascular structures are absent in these gaps but signs of vascular sprouting i.e. neoangiogenesis are evident (B’; arrowheads). **(C)** Semithin sections reveal CAM is still edematous with dilated vessels (asterisks) and regions showing distinct differences in capillary density: lower density with large gaps (asterisks) between capillaries vs. regions of higher/normal capillary density (C’). **D)** The neoangiogenic compartments are determined at the ultrastructural level with epithelium (Ep) detached from the capillaries (black arrowheads; *i*) with some endothelial cells with large vacuoles remaining (asterisks). Signs of endothelial cell (En) activation are observed as philopodia-like projections (arrows) into the lumen and abundance of intracellular organelles, mainly mitochondria (*ii*). Large, rounded, organelle rich pericytes (Pr) are also present but not completely attached to the endothelium. **(E)** In contrast, regions with high vascular density are associated with thickened epithelium with large electron-lucent regions (stars) in tight contact with endothelium. The microvessels revealed more normal structure but still comprised of dense and large endothelial cells (En) often with intraluminal projection at the site of intercellular junction (*i*) and tight peri-endothelial (Pr) contacts (*ii*). Scale bar in A and B is equivalent to 500 μm, 250 μm in B’ and 1 μm in Di, Dii, Ei and Eii.

By 24 hrs post-IR, there was almost complete reperfusion of microvasculature across the peak-irradiated regions (Fig. 4A) with connections between adjacent valley regions and to larger supplying vessels (Fig. 4B). Some gaps in the microvascular network were still observed in the beam path but with many vessel sprouts visible indicative of active neoangiogenesis (Fig. 4B’). The thickness of the mesenchyme was reduced relative to that observed at 1 h post-IR; however some vessels remained dilated (Fig. 4C). A repeated pattern of differential epithelial and endothelial changes could be observed as alternating regions of attenuated epithelium with low capillary density and a thickened epithelium with a higher capillary density (Fig. 4C’). Further characterization at the ultrastructural level revealed regions with attenuated epithelium retracted from the endothelium, which was comprised of enlarged endothelial cells with occasional vacuoles (Fig. 4D). The endothelium was, however, continuous without gaps or disruptions and endothelial cells showed signs of activation as characterized by microvilli-like projections and an abundance of mitochondria (Fig. 4D*i*) as well as robust inter-endothelial contacts with protrusions at these junctions (Fig. 4D*ii*). Rounded, activated, organelle-rich, peri-endothelial cells were observed near the vessel without complete attachment. Regions of the CAM with a thickened epithelium (Fig. 4E) had capillaries comprised of more mature but still enlarged, activated endothelial cells that formed robust inter-endothelial contacts (Fig. 4E*i*). These endothelial cells showed signs of expansion with thin, elongated extensions in close contact with the epithelium and with intraluminal projections at the extending front (Fig. 4 E*ii*).

### Synchrotron minibeam irradiations at 0.05 Gy/s

**Fig. 5:**
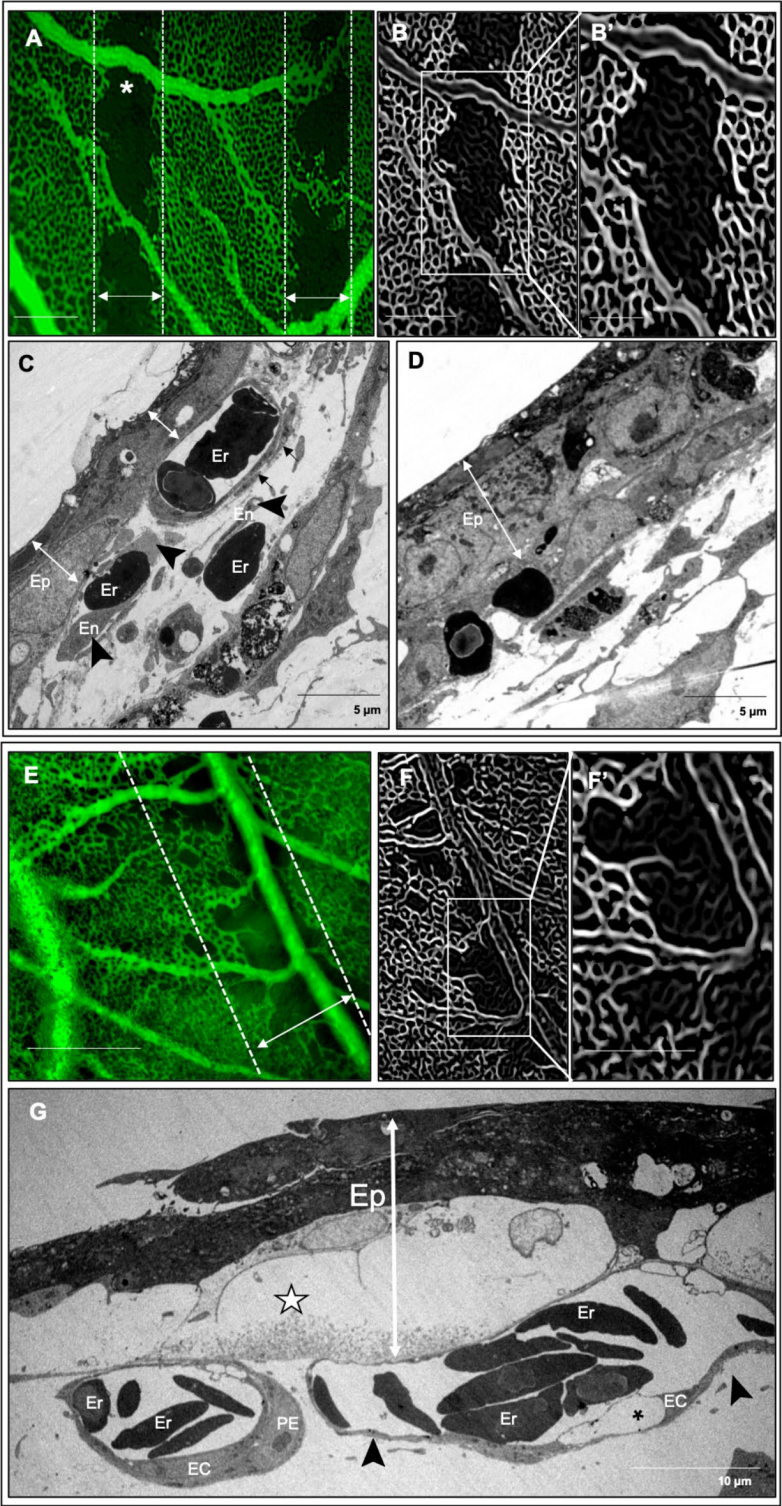
Delivery of 10 Gy peak doses at the lower dose rate of 0.05 Gy/s delays vascular recovery following irradiation. Differential vascular dynamics observed at 7 hours (A, B,C, D) and 24 hours (E, F, G) post-irradiation. **(A)** After irradiation at 0.05 Gy/s, 10 Gy peak doses resulted in sustained loss of perfusion in peak regions at 7 hours post-irradiation. **(B)** Enhancement of vessel connectivity displays a clear distinction between peak and valley regions with a lack of microvascular connection to large vessels (B’). **(C)** Ultrastructural analysis reveals that some CAM regions have destroyed vessels with extravasated erythrocytes (Er) in the mesenchyme in close proximity to unconnected endothelial cells (En; arrowheads). Some vessels remain intact but engorged with Er and with disrupted pericyte coverage (arrows). The epithelium (Ep) is of variable thickness (white double-headed arrows). **(D)** Other CAM regions show complete vascular collapse at the ultrastructural level with no vascular structures present. **(E)** By 24 h post-irradiation, microvasculature rarefaction is observed in the beam path (double-headed white arrow). **(F)** Enhanced visualization of vessel structures clearly depicts the rarefied regions in this region with loss of connection to large supplying vessels **(F’)**. **(G)** Ultrastructural changes at 24 h reveal both restored capillaries with continuous endothelial (En) and peri-endothelial (Pr) coverage as well as capillaries lacking pericyte coverage (arrowheads) with vacuolated endothelial cells (En; asterisks). The epithelium is not homogenous and has grossly dilated regions (double arrowhead) containing large, electron lucent features (star). Scale bar in A, B, E and F is equivalent to 500 μm and 250 μm in B’ and F’.

When irradiated with the reduced, standard clinical dose rate of 0.05 Gy/s, temporal changes in vascular dynamics followed the same pattern as those described following minibeam irradiation at 10 Gy/s. Permeability was observed in the beam path by 1 hr post-IR (Supplementary Fig. S3) and ceased by 2 hrs with an absence of perfusion in these regions as described for 10 Gy/s. However, unlike at 10 Gy/s, occlusion of the microvasculature in the beam path was prolonged and was still present by 7 hrs post-IR (Fig. 5A). There was reduced vessel connectivity between the peak and valley regions (Fig. 5B) as well as to larger supplying vessels that traversed the peak-irradiated region (Fig. 5B’). These differences could be characterized further at the ultrastructural level as condensation of the mesenchyme and destruction of small vessels. This was observed as extravasated erythrocytes in close contact with individual, contracted epithelial cells (Fig. 5C). Collapsed vessels were also observed in some regions as single erythrocytes found amongst compacted, subepithelial tissue (Fig. 5D). After 24 h post-IR, rarefaction of microvasculature in the peak regions was observed (Fig. 5E) and a lack of connectivity of microvasculature to larger supplying vessels in this region and no signs of vascular sprouting (Fig. 5F, F’). At the ultrastructural level, the epithelium remains dramatically enlarged with intact capillaries that show complete endothelial and peri-endothelial coverage and others with attenuated endothelium with large vacuoles and an absence of peri-endothelial cell attachment (Fig. 5G).

### Clinical minibeam irradiations

**Fig. 6:**
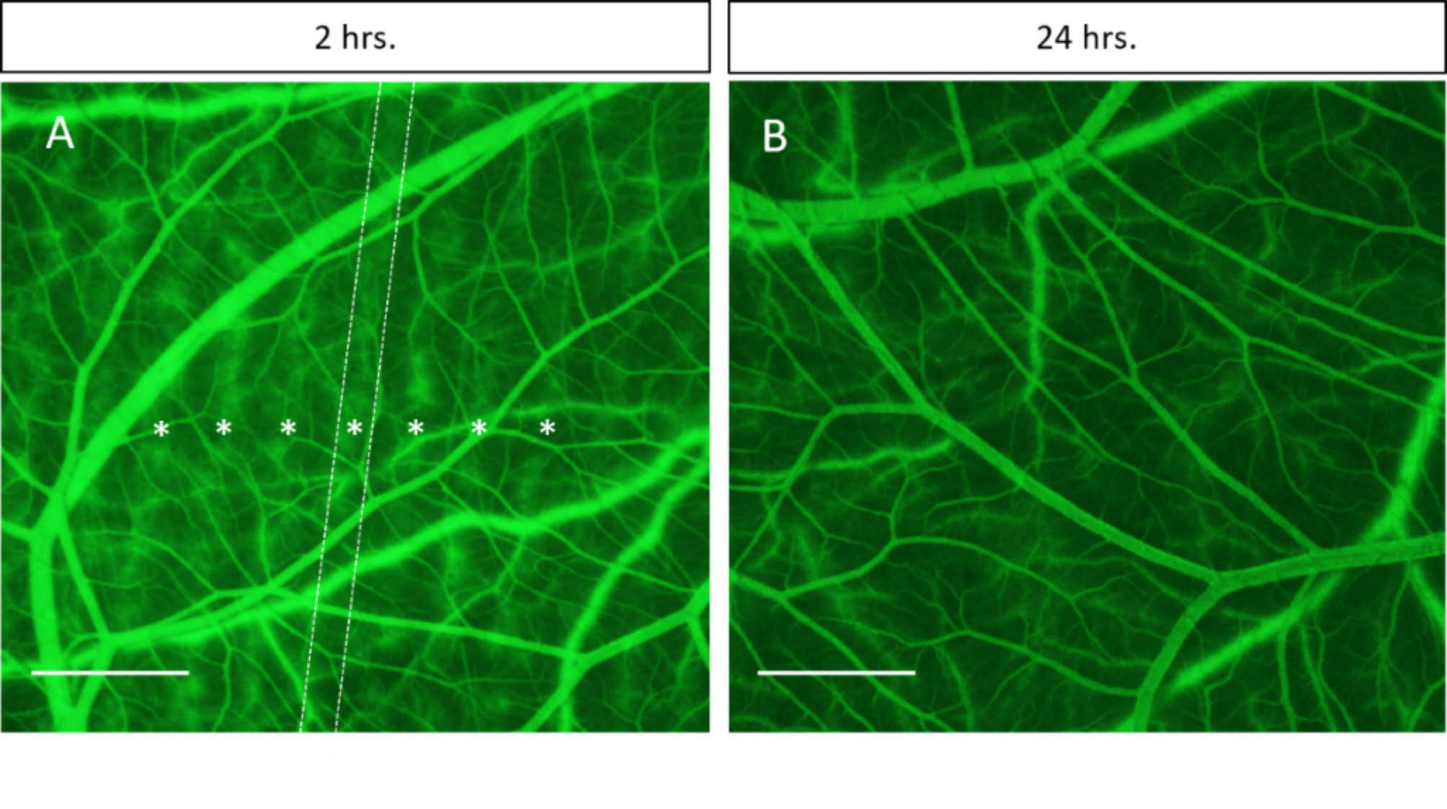
Vascular permeability can be induced by minibeam irradiation delivered with a clinical orthovoltage system. Minibeam irradiation delivered from a clinical X-Strahl 300 orthovoltage unit outfitted with a collimator of the similar spatial geometry of 0.5 mm-wide minibeams spaced 1.1 mm confirmed synchrotron sources are not required to induce reversible vascular permeability. **(A)** Permeability was visible by 2 h post-irradiation in the peak regions  (asterisks). One representative peak region is highlighted by a dotted line for clarity. **(B)** Normal microvascular perfusion was restored by 24 h following irradiation indicating vascular disruption did not lead to progressive disruption of vascular function. Scale bar is equivalent to 250 μm.

Despite the successful induction of vascular permeability with minibeams delivered at dose rates far below those used to initially describe the phenomena with microbeams, physical characteristics still vary between synchrotron sources and clinical irradiators. Therefore, a preliminary proof-of-concept investigation was conducted using a clinical orthovoltage irradiator to deliver minibeam irradiation with the same geometry and peak dose. Vascular permeability was successfully induced with this clinical source, with the characteristic exudation of FITC-dextran (150 kDa) appearing in the peak-irradiated regions 2 h post-IR; a delay in onset compared to the synchrotron irradiations (Fig. 6A). It was also confirmed that this induction of permeability was transient and reversible without inducing widespread, progressive vascular damage as seen by the absence of permeability or vascular occlusion 24 h post-IR (Fig. 6B). These results suggested that synchrotron-like vascular permeability effects may be achieved using widely available clinical orthovoltage sources.

## Discussion

Microbeam SFRT has been shown to induce transient vascular permeability localized to peak dose regions without compromising overall vascular integrity. This “permeability window” has been hypothesized to improve drug delivery to tissue sites with complex vascular barriers such as tumor and brain. This phenomenon has been demonstrated in a glioblastoma model where administration of a chemotherapeutic agent following microbeam delivery enhanced treatment efficacy^20^. The potential of enhancing drug uptake in tumors is of great clinical interest as the aberrant tumor vasculature hinders treatment efficacy of systemic agents. Microbeam SFRT shows promise in addressing this challenge, however, its clinical application is hindered by its use of kGy/s dose rates, limiting its application to synchrotron facilities. It is uncertain whether the unique biological effects of microbeam SFRT depend on such ultra-high dose rates and microscopic geometry, or if they can be replicated with clinically feasible radiation parameters.

We have shown for the first time that transient vascular permeability, previously only described with microbeam SFRT, can be induced using vastly lower dose rates, peak doses and larger, minibeam geometry with both synchrotron and clinical sources. Using the chick chorioallantoic membrane (CAM) model, we were able to induce transient vascular permeability with these adapted parameters. Its induction, transient nature and overall preservation of vascular integrity appeared to be independent of the dose-rate and dictated by the spatial fractionation itself. However, dose rate-dependent changes were observed in vascular dynamics and recovery patterns.

The initial induction of permeability was likely a passive release of vascular components due to the loss of peri-endothelial cells, disrupted endothelial contacts, and endothelial disintegration observed by 1 hr. post-IR (Fig. 1). Given the 150 kDa size of the FITC-dextran particle, its ability to cross the endothelial barrier is only possible following vascular injury since typical vessels limit perfusion of molecules exceeding 70 kDa^31^ and the CAM vasculature at this developmental timepoint is impermeable to albumin, which is approximately 66 kDa^32^. Therefore, this permeability was induced by the localized damage in the peak dose regions in the CAM. Furthermore, the permeability to a molecule of 150 kDa implies that smaller plasma proteins would also escape vasculature and contribute to the observed induction of edema in the CAM mesenchyme in irradiated regions (Fig. 1C). This loss of plasma resulted in hemoconcentration and congestion of vasculature (Fig. 2C), which impeded blood flow and progressed to angioocclusion^33^ (Fig. 2A). This was exacerbated by the accumulation of platelets along the exposed but intact basement membrane (Fig. 2C). Maintenance of the basal lamina is essential for maintaining microvascular integrity^34^ and provides a scaffold for vascular remodeling and functional recovery of damaged vascular networks. Such recovery could have potentially prompted the later reperfusion of occluded microvasculature in peak regions 7 hrs post-IR at 10 Gy/s (Fig. 3A). Vessel reperfusion following occlusion in the CAM could also be attributed to neovascularization in the capillary plexus or recanalization of existing vessels^35,36^. Given the short time frame it is unlikely that this vascular recovery can be attributed to cellular proliferation but was rather likely the result of recanalization of intact vessels or extension of the perfused vascular bed by migrating endothelial cells^37^. Signs of migration were observed at this timepoint as two migrating endothelial filopodia along regions of denuded, but intact, basement membrane (Fig. 3C). Elongation and migration of resident ECs from both sites of endothelial disruption are primarily responsible for regeneration of the endothelium in a variety of injury models and tissues^38–43^. This is coupled with evidence of endothelial cell activation observed at the ultrastructural level as large, organelle-rich cells with microvilli-like extensions extending into the capillary lumen (Fig. 3D). Such endothelial protrusions are associated with growing capillaries during wound healing^44^ further establishing evidence of vascular repair after minibeam irradiation. The extent of this recovery was observed 24 h post-IR where microvasculature perfusion was restored across the beam path (Fig. 4C), likely as a result of both revascularization and neovascularization at this later timepoint (Fig. 4B’, D, E).

In contrast, the beam path following irradiation at 0.05 Gy/s, remained unperfused 7 h post-IR (Fig. 5A). This prolongation of vascular occlusion in the beam path was associated with ultrastructural changes that revealed vessel destruction in some areas of the microvasculature comprising the CAM’s capillary plexus (Fig. 5C, D). These results mirror those of early studies investigating vascular changes in the CAM following ionizing radiation where such changes following 11 Gy homogenous X-ray irradiation were progressive beginning with plasma loss and hemoconcentration followed by endothelial vacuolization and occlusion of small vessels eventually leading to circulatory collapse^45,46^. Larger areas of microvascular rarefaction in the beam path were observed 24 h post-IR at the 0.05 Gy/s dose rate (Fig. 5E). Despite this, there is evidence at the ultrastructural level that not all capillaries in these CAMs are damaged as both normal and damaged vessels are seen proximal to one another (Fig. 5G). It is likely that the repair capacity is only delayed after irradiation at the lower dose rate but preservation of larger vessels and microvasculature in the valley regions likely contributed to neovascularization in the unperfused peak areas. Overall, damage to peak-irradiated microvasculature is not progressive and vascular integrity is maintained. This may differ in tumor tissue where vessel connectivity is interrupted and vascular networks are dysregulated. It has been shown that vascular occlusion following permeability induced by conventional radiation sources resulted in progressive vascular occlusion and loss of vascular function in tumors exposed to doses exceeding 5 Gy^47,48^. Therefore, there is a clear difference in vascular effects between conventional, homogenous radiation and minibeam radiation.

Furthermore, the successful induction of vascular permeability with a clinical orthovoltage unit outfitted to deliver minibeams of the same geometry at a dose rate of 0.02 Gy/s, confirms this can be achieved in a clinical setting. However, the onset of permeability was delayed, appearing only at 2 h post-IR (Fig. 6A). Similarly, the vascular network appears to be intact and perfused at 24 h post-IR (Fig. 6B). This is possibly attributed to one or more differences between synchrotron and clinical orthovoltage irradiations, which can result in variations in dose distribution and peak-to-valley dose ratio. Factors such as energy spectrum, source size, beam divergence, and irradiation configuration influence the primary beam geometry and the radiation scatter contribution at the target position, affecting the overall dose distribution. To quantify the impact of these factors on vascular permeability, additional experiments are necessary. Further investigation is therefore required to determine the details of the vascular changes associated with permeability induction in a clinical setting and will be the subject of future investigations. This should include investigating the temporal dynamics of this permeability induction in non-embryonic tissue. Although the CAM is a useful model to observe vascular changes in real-time, its embryonic nature may show accelerated progress through stages of damage and repair compared to that in a mature animal. However, the CAM microvasculature and extracellular matrix does retain key features found in the mammalian system^49^ suggesting the permeability induced by minibeams in the CAM microvasculature will likely translate to adult tissues. Furthermore, the differential sensitivity of the microvasculature to ionizing radiation relative to that of larger vessels^50^ is an important feature recapitulated in this model.

Further development in the use of minibeam irradiation with clinical sources to induce transient vascular permeability offers an avenue to introduce minibeam irradiation into a clinical treatment regimen. This approach has the potential to enhance efficacy of systemic agents without compromising healthy tissue integrity. This also provides an avenue to further explore additional biological effects, traditionally only associated with microbeam SFRT, in a clinical setting, promoting its translation from pre-clinical synchrotron studies to clinical application.

## Electronic supplementary material

Below is the link to the electronic supplementary material.

Supplementary Material 1

## Data Availability

All data generated or analysed during this study are included in this published article and its supplementary information files. Raw images can be made available from the corresponding author on reasonable request.
