# Supplementary material for: Spatially fractionated minibeam radiation delivered at clinically feasible dose rates induces transient vascular permeability
Source: Sci Rep. 2025 Mar 10;15:8210. doi: 10.1038/s41598-025-87395-9 (PMC11894116; doi:10.1038/s41598-025-87395-9)
Supplement: Supplementary file 1 — Supplementary Material 1 [file 41598_2025_87395_MOESM1_ESM.docx]

**
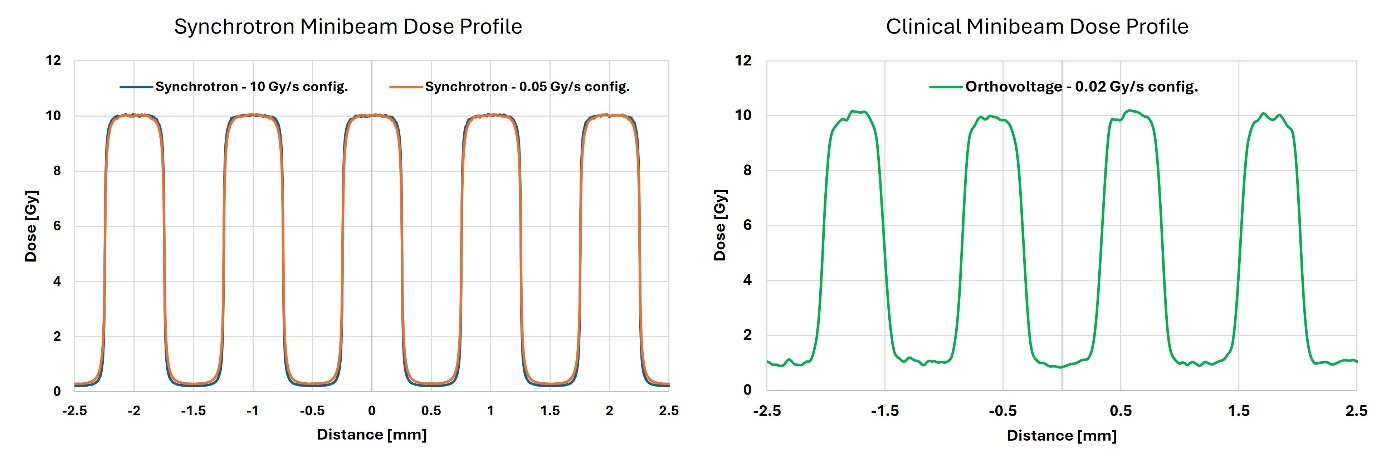
**

B

A

**Figure S1: Dose profiles of synchrotron and clinical irradiaitons.** Dose profiles of 5 central minibeams generated by Monte Carlo simulations at a depth of 2 cm for the synchrotron minibeams (A) and by EBT3 radiochromic film dosimetry for the clinical orthovoltage minibeams (B). For the clinical orthovoltage unit 3 separate measurements were obtained and average peak and valley doses calculated from these 3 measurements and used as the nominal calibration. All films were calibrated using the same beam energy as used for the CAM irradiations (180 kVp).

| Parameter | Synchrotron source at 10 Gy/s | Synchrotron source at 0.05 Gy/s | Clinical Orthovoltage source at 0.02 Gy/s |
| --- | --- | --- | --- |
| Spectrum mean energy [keV] | 162 | 198 | 75 |
| Source to sample distance [m] | 40.8 | 40.8 | 0.31 |
| Source to multislit collimator distance [m] | 39.4 | 39.4 | 0.3 |
| Multislit collimator geometry: aperture width / center-to-center spacing [mm] | 0.5 / 1.0 | 0.5 / 1.0 | 0.5 / 1.1 |
| Peak dose [Gy] | 10 | 10 | 10 |
| Valley dose [Gy] | 0.216 | 0.296 | 1.35 |
| Peak to valley dose ratio (PVDR) | 46.3 | 33.8 | 7.4 |

**Table S1:**  Summary of irradiation parameters used for all radiation conditions described for both the synchrotron and clinical source.

**
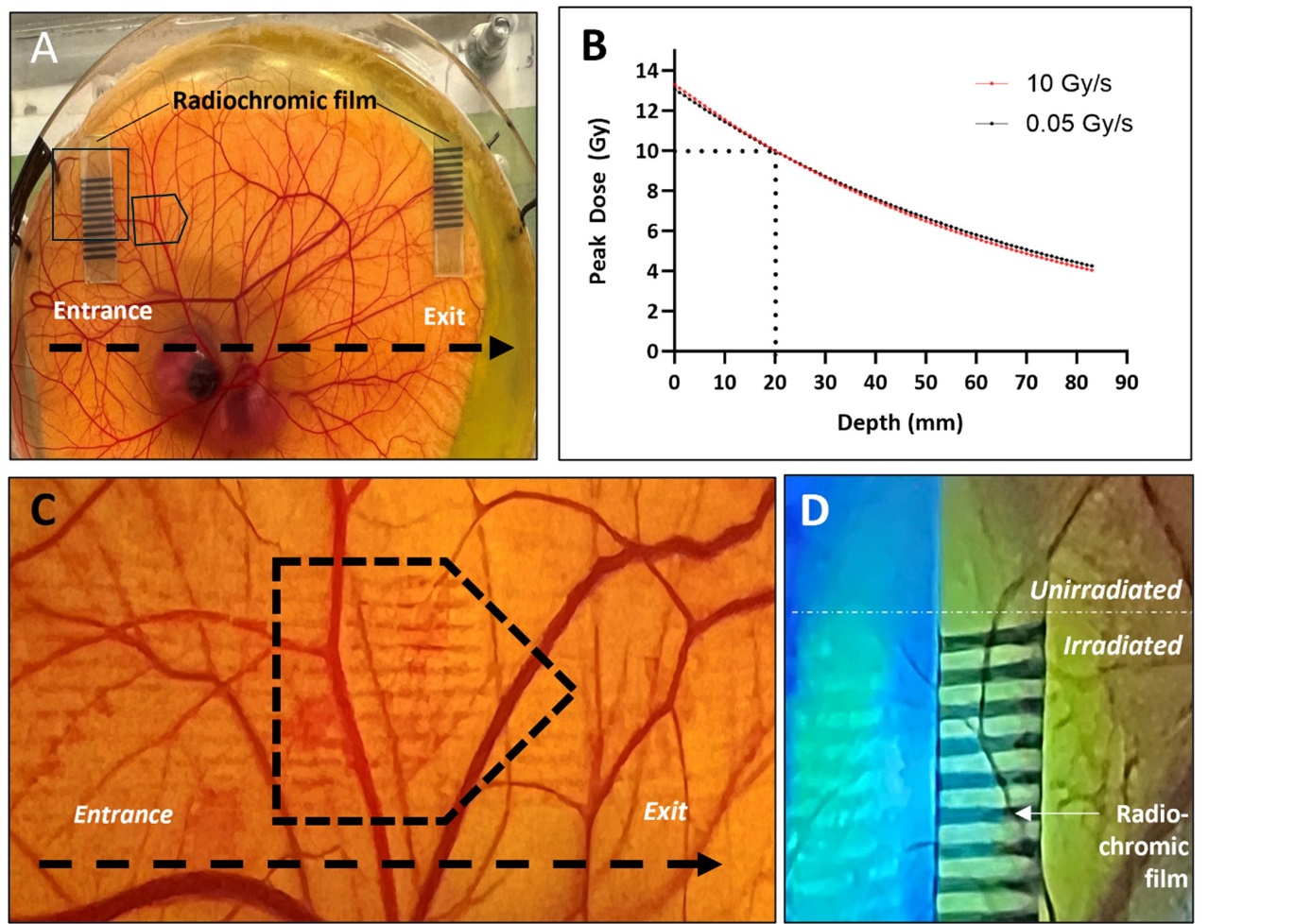
**

**Figure S2: Setup of CAM irradiations**. (A) Radiochromic film was placed on each CAM to mark the entrance and exit of the beam path. This allows for identification of the region of interest which corresponds to peak dose deposition of 10 Gy at 2 cm from the entrance based on the modeled depth dose profile through the CAM (B). This region is excised after fixation as indicated in (C) to preserve directionality when processing tissue for electron microscopy. Pentagon in C corresponds to the pentagon in A. The “minibeams” are visible as a dark red stripes due to the accumulated and fixed red blood cells (RBC) in the beam path. FITC dextran permeability in the beam path can be confirmed macroscopically using a fluorescent stereomicroscope (green stripes) in combination with bright light visualizing the radio-chromic film (D). D corresponds to the rectangle in A.


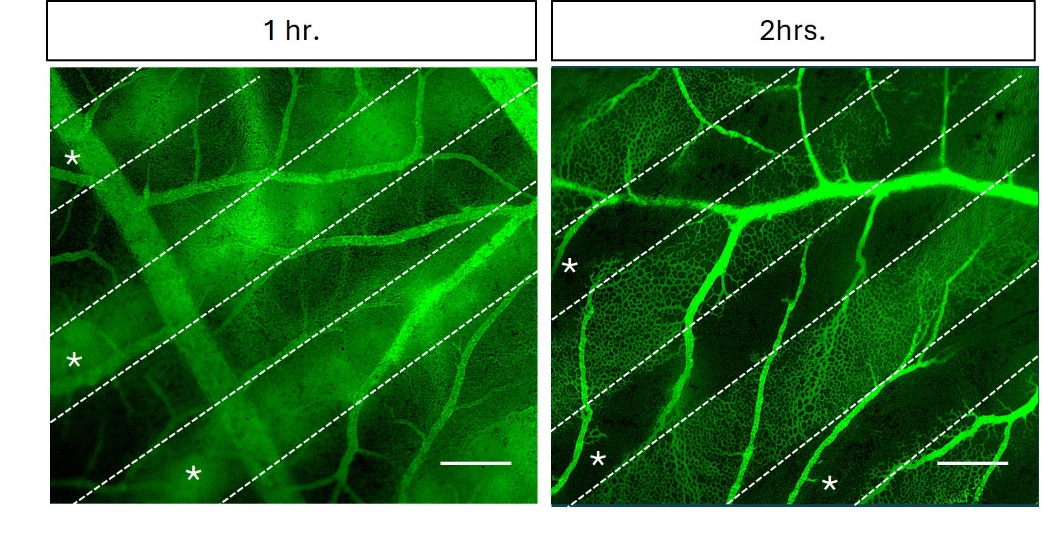


**Figure S3: Permeability dynamics following synchrotron minibeam irradiation delivered at 0.05 Gy/s.** Permeability observed in regions of minibeam peak dose deposition (asterisks) at 1 hour post-irradiation and ceased by 2 hrs. Peak regions remain unperfused at 7 hrs post-irradiation. Scale bars are equivalent to 500 μm.
